# Supplementary material for: Comparative genomics provides new insights into the diversity, physiology, and sexuality of the only industrially exploited tremellomycete: Phaffia rhodozyma
Source: BMC Genomics. 2016 Nov 9;17:901. doi: 10.1186/s12864-016-3244-7 (PMC5103461; doi:10.1186/s12864-016-3244-7)
Supplement: Additional file 6: — List of orphan genes with links to PFAM (related to Additional file 1: Table S1). (ZIP 1428 kb) [file 12864_2016_3244_MOESM6_ESM.zip › PFAM_UNIQUE/G02403_P.html]

#                                                                            --- full sequence --- -------------- this domain -------------   hmm coord   ali coord   env coord
  
# target name        accession   tlen query name           accession   qlen   E-value  score  bias   #  of  c-Evalue  i-Evalue  score  bias  from    to  from    to  from    to  acc description of target
  
#------------------- ---------- ----- -------------------- ---------- ----- --------- ------ ----- --- --- --------- --------- ------ ----- ----- ----- ----- ----- ----- ----- ---- ---------------------
  
G02403\_P             -           1325 SURF2                PF05477.6    244       0.8    7.4   5.5   1   2   0.00071      0.43    8.3   2.0   132   207   403   483   397   512 0.53 - Surfeit locus protein 2 (SURF2)  
G02403\_P             -           1325 SURF2                PF05477.6    244       0.8    7.4   5.5   2   2       3.2   1.9e+03   -3.6   0.1   109   143   731   765   726   775 0.62 - Surfeit locus protein 2 (SURF2)
